# Factors affecting HIV prevention communication between professional nurses and high school learners in eThekwini

**DOI:** 10.4102/hsag.v29i0.2364

**Published:** 2024-05-08

**Authors:** Thina S. Ngidi, Nongiwe L. Mhlanga, Thinavhuyo R. Netangaheni

**Affiliations:** 1 Department of Health Studies, College of Human Sciences, University of South Africa, Pretoria, South Africa

**Keywords:** communication, eThekwini, HIV prevention, learners, professional nurses

## Abstract

**Background:**

Communication within healthcare settings is crucial for a therapeutic relationship between nurse and patient especially in preventing human immunodeficiency virus (HIV) among adolescent girls. The school health programme in South Africa provides an opportunity to provide HIV prevention services among adolescent girls, however, uptake is low and effective communication can address this.

**Aim:**

The study’s purpose was to describe factors affecting HIV prevention communication between professional nurses and high school learners in eThekwini Metropolitan Municipality.

**Setting:**

The study was conducted in two high schools in eThekwini Metropolitan Municipality.

**Methods:**

The study used a qualitative approach and a descriptive research design. A semi-structured interview guide was used to conduct face-to-face interviews with 30 participants after ethical approval from the researchers’ affiliated institution and informed consent from participants or their guardians were obtained. Thematic analysis was used to analyse the data.

**Results:**

Participants were aged between 14 years and 17 years. Most (25) participants were female. Three main themes emerged from the study: communication between the school health nurses and high school learners is facilitated by trust, communication is hindered by fear, and infrequent visits by nurses affect communication positively or negatively.

**Conclusion:**

Health education on HIV is essential to prevent HIV among adolescent girls. Effective communication between healthcare providers and adolescent girls facilitates such health education.

**Contribution:**

The uptake of HIV prevention services by adolescent girls can be improved by communication that facilitates the therapeutic relationship which is founded on trust, with frequent visits by nurses.

## Introduction

Communication in healthcare settings plays a critical role in enabling the therapeutic relationship between nurses and patients of all ages. Communication is a two-way process that enables the exchange of information using common signs, behaviour, or symbols, and includes numerous components such as the sender, the receiver, and the message and the feedback (Alshammari, Duff & Guilhermino 2019). Effective communication between nurses and patients plays a critical role in ensuring the patients have a positive perception of the treatment process as well as the outcomes of the treatment (Alshammari et al. 2019). Healthcare workers must communicate effectively to improve patients’ satisfaction and health outcomes (Al Ghunimat et al. 2020). In this context, the communication is between the professional nurses and learners in high school who use the school health programme where the expected outcome of healthcare is the usage of human immunodeficiency virus (HIV) prevention services for the reduction in HIV transmission among adolescents.

The importance of effective communication when working with adolescents is illustrated through the HIV challenges among adolescents and young people in South Africa. In South Africa, there are approximately 20% of all people living with HIV, as well as 20% of those newly diagnosed with HIV infections, rendering the country the largest epicentre of the HIV pandemic globally (Allinder & Fleischman 2019). About 4500 South Africans are presenting with new infections every week, while 60% of women are affected or infected with HIV in some communities of KwaZulu-Natal province (Allinder & Fleischman 2019). In addition, one-third of those infected with HIV were adolescent girls and young women (AGYW) aged 15 years to 24 years (Allinder & Fleischman 2019). This age group includes junior and senior high school learners; that is, Grades 9 to 12 whose expected age is between 14 years and 17 years in South Africa. In addition to a higher prevalence of HIV, adolescent girls in South Africa also acquire HIV 5 years to 7 years earlier than their male counterparts, with a three-to-four-fold higher incidence (Pettifor et al. 2019).

To address the above problem, the Department of Basic Education (DoE) and Department of Health (DoH) in South Africa developed the Integrated HIV and AIDS services policy for schools in 2012 which comprises a package of onsite services by school health nurses that include treating minor ailments such as skin conditions and sexual and HIV health services where required. Such a policy focusses on dual protection services for preventing pregnancy, sexually transmitted infections (STIs) and HIV infection, as well as the provision of HIV Counselling and Testing (HCT) (Allinder & Fleischman 2019). A referral system was also designed to ensure complete management of any condition that may or may not be HIV-related. To provide services in accordance with the policy, teams of school health nurses visit schools to provide the services and also provide follow-up services where referral cannot be done (Allinder & Fleischman 2019). Justifying the initiative to provide school health services, Ethier (2019) proffered that schools play a highly important role in promoting health and safety, and preventing HIV among youth who spend most of their day in school, through the provision of knowledge opportunities, skills, and resources needed to prevent HIV during adolescence and into adulthood. Quality health education, including primary HIV prevention connected to healthcare services and school environments, supports youth and helps them feel connected and safe ensuring their academic success (Ethier 2019).

Despite the existence of HIV prevention services for learners in high schools in South Africa, the uptake of HIV testing services among adolescents remains low (Lebina et al. 2019). One of the reasons for the low uptake of HIV testing services is a low HIV risk perception among adolescents (Muravha et al. 2021). These low-risk perceptions among high school learners and the overall high incidence of HIV infection among AGYW underscore the importance of increasing usage and access to HIV prevention services provided in schools. Therefore, good environments should be created where learners can receive HIV education on prevention during adolescence and into adulthood (Kharsany et al. 2018).

One of the means to foster a good environment to ensure the utilisation of HIV prevention services in school health programmes is effective communication. A South African study that sought to describe the perceptions of adolescents on adult influence on HIV programmes concluded that adolescents required effective communication to support decisions on HIV prevention and reproductive health (Bergam et al. 2022). Another study conducted in the United States of America (USA) also reiterated the issue of effective communication in providing HIV prevention services to young women and adolescents. Zelazny et al. (2019) found that young women valued effective communication with healthcare workers to discuss sensitive information. To enable effective communication, young women need to be in ‘safe spaces’ created by the healthcare workers (Zelazny et al. 2019). Communication required when providing health education for young women should ensure that the healthcare provider speaks to the adolescents and young women ‘as friends’ (Zelazny et al. 2019).

The issue of young women wanting to be treated as friends in healthcare settings when communicating with nurses is reiterated by Adams et al. (2017) who found that fear was an issue among adolescents seeking health education on sensitive issues like HIV. The fear emanates from the stigma attached to HIV itself (Adams et al. 2017). In addition to creating safe spaces for communication and eliminating fear, communication should also be done at the appropriate time and space (Zelazny et al. 2019). Adolescent girls and young women prefer to communicate about sensitive issues with healthcare workers during a consultation visit rather than at the start of the consultation (Zelazny et al. 2019).

A study in Fiji found that good patient-centred communication facilitates a therapeutic relationship; however, sometimes communication in healthcare is affected by the attitude of the healthcare workers (Chandra & Mohammadnezhad 2021). The poor attitude that negatively affects patient-centred communication is characterised by rudeness, healthcare workers not responding and/or talking without respect (Chandra & Mohammadnezhad 2021). As such, effective communication that is built on respect for patients also fosters trust in the healthcare system and improves satisfaction with the services provided (Negro et al. 2020). To enable such effective communication between nurses and high school learners, a Nigerian study found that nurses themselves have to be trained to provide effective communication on HIV prevention regardless of their beliefs (Salau & Ogunfowokan 2019). Such communication can be aided by audio-visual materials which high schools can provide (Salau & Ogunfowokan 2019).

Patient-centred communication aims to create a trusting relationship between the patient and the healthcare provider (Wolderslund, Kofoed & Ammentorp 2021). In addition, when communication is patient-centred, the healthcare provider tends to demonstrate empathy towards the patient (Wolderslund et al. 2021). Communication between young women and healthcare providers should ensure that young women and adolescents feel cared for by healthcare workers (Zelazny et al. 2019). Furthermore, the factors affecting communication among young people and nurses include intergenerational pressures from caregivers, teachers and healthcare workers who often stigmatise choices made by adolescents regarding their sexual behaviour which affects the patient-centred communication patterns between young people and healthcare workers (Bergam et al. 2022).

The Relationship: Establishment, Development and Engagement (REDE) model of healthcare communication developed by Windover et al. (2014) formed the theoretical framework for this study. The model describes three phases in the relationship between healthcare providers and patients. These phases are establishment, development, and engagement. In establishing the communication relationship, the healthcare provider respects the client and collaboratively sets the agenda for the visit. With high school learners, this entails open-ended questions on their concerns about reproductive health and HIV. The second phase is the development phase, whereby reflective listening skills are used and patient narratives are sought (Windover et al. 2014). The last phase is the engagement phase where the diagnosis news is shared and a collaborative plan is developed (Windover et al. 2014). With high school learners in school health services, this may entail informing them of their HIV test results.

By fostering good patient-centred communication, it is expected that high school learners would have trust in the school health services and increase their usage of HIV prevention services. Therefore, this study aims to describe the factors affecting HIV prevention communication between professional nurses and high school learners in eThekwini Metropolitan Municipality.

## Research methods and design

The study used a qualitative approach, which is concerned with obtaining insight into the factors affecting HIV prevention communication between professional nurses and high school learners in eThekwini Metropolitan Municipality. The study further adopted a descriptive qualitative study design, which is a comprehensive summary of phenomena in everyday language not rooted in phenomenology, ethnography, or grounded theory (Polit & Beck 2020).

The study was conducted in two high schools from the eThekwini Metropolitan Municipality. The eThekwini Metropolitan Municipality is one of the 11 districts in KwaZulu-Natal, a province in which HIV prevalence is highest in South Africa. Of note, the researchers provide high school health services in the eThekwini Metropolitan Municipality and interact with the study participants in their natural settings.

The two schools in eThekwini Metropolitan Municipality were selected conveniently. Non-probability sampling was applied to choose eligible participants, who were purposively selected. The purposive sampling strategy assumes that the researchers’ knowledge or judgement of the research population and its dynamics is an enabler for selecting individuals in the sample (Holloway & Galvin 2017). In this study, the researchers purposively made a judgement of learners between the ages of 12 years and 19 years who were adolescents at risk of HIV infection and utilised HIV prevention services.

### Inclusion and exclusion criteria

The target population was all learners at the two schools eligible for HIV prevention services. In light of this, the following inclusion and exclusion criteria were applied in the study.

#### Inclusion criteria

- A learner registered in any of the two identified high schools who had directly experienced the HIV prevention services from the school health programme.
- A female or male learner between the ages of 12 years – 19 years.
- Willingness to participate in the study.

#### Exclusion criteria

- A learner not willing to take part in the study or whose guardians did not consent to their participation in the study.
- Learners who were ill and could not make decisions, or weigh the risks and benefits of participation.

A total of 30 study participants were selected from the population and this sample size was determined by data saturation which is the point where additional recruitment of study participants was not generating new data (Grove, Burns & Gray 2020). Table 1 shows the distribution of participants.

**TABLE 1:** Distribution of the participants.

Study setting	Number of participants
First high school	15
Second high school	15

**Total**	**30**

### Ethical considerations

Ethical approval to conduct the study was obtained from the Human Sciences Research Ethics Review Committee (Reference number 63309386_CREC_CHS_2022). The researchers requested and obtained permission to undertake the study from University of South Africa’s (UNISA) Research Ethics Committee and the KwaZulu-Natal Provincial Department of Education’s eThekwini district management for the involvement of the selected high school learners in this research. The researcher further understood the requirement for the study’s compliance with Section 73 of the *National Health Act (No. 61 of 2003)* which stipulates that health institutions should have institutional review boards that grant permission for health research at facilities; therefore, permission was sought from the district management. The researchers outlined the expected level and nature of the participants’ involvement, including the rights they are entitled to, and this outline was further interpreted in their home language of isiZulu (Kumar 2020).

After fully disclosing the study, each participant was provided with an informed consent form to sign if they were 16 years or above, or for their guardians to consent on their behalf if they were below 16 years of age. The researchers also acknowledged that the Integrated School Health Programme includes an assent form for learners over 12 years who can consent without their parents’ permission; however, for purposes of the study, consent was sought from guardians of participants below 16 years. The participants were informed that they could withdraw from the study whenever they felt uncomfortable. In addition, the researchers ensured that the study participants encountered no form of harm. To ensure confidentiality and privacy, interviews were conducted in a private room and information about the research was kept in a locked cupboard and a password-protected computer. The researchers also used pseudonyms to ensure anonymity. Furthermore, to abide by the *Protection of Personal Information Act* (POPIA), and maintain confidentiality, no research information was shared to anyone who was not part of the research team (POPIA 2013).

### Data collection

In-depth semi-structured interviews were used to obtain information on factors affecting communication between professional nurses and high school learners. This enabled a better understanding of individual participants’ perspectives. Interviews were conducted at the schools at the onsite service centres. In this regard, the researchers prepared an interview guide to direct the proceedings during interviews. One ‘grand tour’ question was used and this question was ‘Describe HIV prevention communication between the nurses and learners during the provision of health education?’ to prompt them to express their views and experiences spontaneously. Depending on the participants’ responses to the ‘grand tour’ question, probing or sub-questions were applied as follow-up for clarity or further input in line with the study objective and research problem (Grove et al. 2020). A field notebook was used to document observations of the participants’ non-verbal behaviour and communication during the interviews (Creswell 2020). Furthermore, the researcher used an audio-recorder with the participants’ concurrence, to ensure that none of the participants’ interview information was omitted, lost, or missed (Efron & Ravid 2019).

### Data analysis technique

To analyse the data, Tesch’s eight-step procedure was used (Creswell 2020). The first step is transcribing the data, followed by a second step of reading and understanding the meaning of the transcribed data. The third step entailed coding the data according to the regularity of concepts which was followed by the fourth step of grouping the categories of concepts. The fifth step grouped these categories into codes. The sixth step of reviewing the consistency of developed codes was followed by a seventh step of deriving meaning from the developed codes. The last step presented the findings.

### Establishing trustworthiness

To ensure the study’s trustworthiness, the researchers applied and documented all research processes and methods. A pre-test of the semi-structured interview guide was also done with three participants to ensure the research objectives were met through the in-depth interviews. After the pretest, no adjustments were made to the research instrument and the outcomes of the pretest were not used in the final analysis.

To ensure trustworthiness, the criteria by Lincoln and Guba 1985 were used (Polit & Beck 2020). Credibility was ensured by confirming the study findings with the high school learners who participated in the study. The researchers ensured dependability by asking peer researchers to check the results of the analysed data, to ensure the researchers’ subjective bias was not reflected. The researchers also took into account how their personal experiences may influence the responses and allowed participants to lead the discussions to ensure confirmability. A description of the study setting, characteristics of participants and study methods has been provided to enable transferability.

## Results

Data were collected from 30 high school learners from two high schools in the eThekwini Metropolitan Municipality. The majority of the participants were female (83%) while 7% were male. The females were more open to participating in the research as most of them access HIV prevention services in comparison to the males. The males who declined expressed shyness and fear of participating. The most represented grade was Grade 10 (40%), followed by Grade 9 (27%), then Grade 11 (20%) and lastly Grade 8 (13%). Table 2 shows the sample demographic characteristics of the participants.

**TABLE 2:** Overall biographic data for all participants.

Participant	Age (years)	Gender	Grade
1	16	F	10
2	16	F	10
3	15	F	10
4	16	F	10
5	16	M	10
6	16	F	10
7	15	F	10
8	17	F	10
9	14	F	9
10	16	F	9
11	15	F	9
12	15	F	9
13	14	F	9
14	16	F	9
15	16	F	9
16	15	F	10
17	16	F	10
18	15	F	10
19	15	M	10
20	14	M	9
21	17	F	11
22	16	M	11
23	17	M	11
24	17	F	11
25	16	F	8
26	16	F	11
27	17	F	11
28	14	F	8
29	14	F	8
30	15	F	8

P, Participant; M, Male; F, Female.

### Themes emerging from the study

Three themes emerged from the study. The first theme was that HIV prevention communication between the school health nurses and high school learners is facilitated by trust. The first subtheme to emerge from this theme one was that trust is affected by familiarity with the nurses which impacts how learners communicate with school health nurses. The second theme was communication is hindered by fear. The subtheme to emerge from theme two was that different ages of nurses and learners create fear which hinders communication. The third emerging theme was less frequent visits by nurses affect communication. The third theme was supported by two subthemes: less frequent visits positively affected communication and less frequent visits negatively affected communication.

### Theme 1: HIV prevention communication between school health nurses and high school learners is facilitated by trust

The theme of HIV prevention communication being facilitated by trust between the learners and the school health nurses was described by the participants. Participants detailed that trust played a pivotal role in shaping their interactions with school health nurses. They emphasised that when the trust was established, communication flowed effectively; conversely, when trust was lacking, communication suffered. Participant 21 noted that they trusted the school health nurse as such communication was good. The participant further stated that trust was also facilitated by the school health nurses keeping their promises. Some participants noted that there was no trust between them and the school nurse which made communication poor. Participants 23 and 17 noted the issue of trust facilitating communication by noting that communication was poor because of the lack of trust. The quotes from the participants to support the theme of trust enabling communication are shown below:

‘The nurses’ attitude is bad in a way that you don’t know whether to trust them or not, but I guess it depends on the individual. I don’t trust them. “At times you use your friend’s experience and have your attitude”.’ (16 year old, female, Grade 10)

‘Communication is not good because we don’t trust easily.’ (17 year old, male, Grade 11)

‘Communication is good. I trust the nurses … School health nurses are friendly, and they teach us well about health issues. They answer our questions politely. They keep their promises when you request a service from them.’ (17 year old, female, Grade 11)

Of note, females (Participants 21 and 17) and male (Participant 23) participants described the issue of trust facilitating communication in the school health programme. Two different grades described the issue of trust enabling communication in the school health programme.

#### Subtheme 1.1: Trust is affected by familiarity with the nurses which impacts how learners communicate with school health nurses

A sub-theme to emerge from the first main theme was that trust is also affected by familiarity with the nurses which affects communication in using school health HIV prevention services. In the descriptions of trust being affected by familiarity, which then determines communication, participants noted that if there was familiarity, they would not trust the school nurses which made communication difficult. The issue of familiarity with the nurses was described by Participants 7, 19, and 18 who shared that since the nurses were from the communities that learners lived in, it was difficult to communicate with them as there was familiarity. The quotes to support this subtheme are shown below:

‘Communication is complicated; some nurses know us from the community.’ (15 year old, female, Grade 10)

‘Some nurses are known to my family. Some of their attitudes are spiteful because they know you and you end up not getting the wanted services because you don’t know if they will tell on you.’ (15 year old, female, Grade 10)

The participants who described the issue of familiarity were all in Grade 10 and were all aged 15 years. Two of these participants (Participant 7 and 18) were female while one was male (Participant 19).

### Theme 2: Communication is hindered by fear

The second theme to emerge was that communication is hindered by fear of the nurses and parents. Both positive and negative issues of fear were described by the participants. However, the major impediment to communication because of fear was the fear of the attitude of the nurses. The fear of nurses because of their attitude was described by Participants 1, 4, 9 and 15, while one participant (Participant 25) described the fear of their parents.

The issue of fear hindering communication was expressed by Participant 9 who noted that the learners were afraid of nurses. Participant 1 also expressed the issue of fear of HIV testing, because of the nurses’ attitude. The nurses’ bad attitude causing fear to test for HIV was also stated by Participant 4 who noted that they would not communicate with the nurses because of the fear of the nurse and their attitude. The quotes below show the issue of communication being hindered by fear of parents, HIV, and the nurses:

‘The nurses’ attitude is disgusting, and you are scared to go for HIV testing.’ (16 year old, female, Grade 10)

‘Some nurses have bad attitudes; they are not patient with us. You become scared and just walk away from them.’ (16 year old, female, Grade 10)

‘Learners are afraid of nurses, communication is complicated.’ (14 year old, female, Grade 9)

‘Some nurses are really bad; they will shout at you for no particular reason, I am scared of them shame.’ (16 year old, female, Grade 9)

Notably, all the participants who described the issue of fear of parents, HIV, or nurses were females. Five of these participants were 16 years old, while one was 14 years old (Participant 9).

#### Subtheme 2.1: Differences in the age of nurses and learners create fear which hinders communication

The first subtheme to emerge from Theme 2, on the issue of fear was that the fear of nurses is exacerbated by the age differences between the learners and the school nurses which ultimately deters the learners from accessing HIV prevention services from the school health programme. In their descriptions, the participants stated that the nurses were old or the nurses themselves perceived that the learners were too young to be accessing HIV prevention services which in turn determined how they communicated. Participant 11 spoke at length on the issue of the age difference between the learners and the school nurses by noting that the nurses perceived the learners as too young to be testing for HIV, while, on the other hand, the learners themselves failed to communicate with the nurses because they were old. In addition, Participant 25 reiterated that the nurses felt the learners were young to be accessing HIV testing services. Participant 5 noted they had experienced good communication after interacting with a young nurse in the school health programme. The expressions from the participants are shown below:

‘Nurses attitude contributes a lot. The comments that they make, like if I want to test, they will say “But you are too young to test” You then decide there and there to never go back. I think with communication it’s bad because nurses who come to school are old.’ (15 year old, female, Grade 9)

‘They say we are young to test; they always make these comments about testing while you are a learner. They don’t give us any sympathy at all.’ (16 year old, female, Grade 8)

Regarding the gender of the participants who noted the issue of communication being affected by the differences in ages between the learners and the nurses, most of the participants were female while one was male (Participant 5). Also, the issue of different ages was expressed by participants of all grades – Grade 8 (Participant 25), Grade 9 (Participant 11), and Grade 10 (Participant 5).

### Theme 3: Less frequent visits by nurses affect communication

The third theme to emerge was that less frequent visits by the nurses affect communication. From their descriptions, the participants shared that the nurses did not visit their schools often and this affected the communication. Two subthemes supported the theme that less frequent visits affect communication. The first subtheme was less frequent visits positively affected communication and the second subtheme was less frequent visits negatively affected communication.

#### Subtheme 3.1: Less frequent visits positively affected communication

Some participants highlighted that these less frequent visits made it easier for them to communicate with the nurses. Participant 2 shared their opinion on the issue of less frequent visits by noting that although there were infrequent visits by nurses, communication remained good between the school health nurses and the learners:

‘The communication is really good because they came one day to offer Covid-19 vaccinations.’ (16 year old, female, Grade 10)

Participant 11 reiterated that the less frequent visits by the nurses fostered good communication by comparing nurses with the teachers:

‘I think it important because students are more likely to speak to a nurse who is not always at school rather than teachers who may not take the problem seriously.’ (15 year old, female, Grade 9)

#### Subtheme 3.2: Less frequent visits negatively affected communication

Some participants shared that the less frequent visits by the nurses resulted in poor communication. The issue of less frequent visits by the nurses affecting communication was described by Participant 1 who shared that the school nurses do not visit frequently. Participants 13 and 14 also noted that the nurses are not available, and as such there is no communication with them. The quotes to support the subtheme are shown below:

‘They don’t come here often as I have never seen them.’ (16 year old, female, Grade 10)

‘I don’t think there is any communication, nurses rarely come to school.’ (14 year old, female, Grade 9)

‘What I would like to say about that, is that I am quite not sure because I have never interacted with a nurse while at school. I won’t say much.’ (16 year old, female, Grade 9)

The participants who described the less frequent visits were all female, with three of them in Grade 9 (Participants 11, 13 and 14) and two (Participants 1 and 2) in Grade 10. Regarding their ages, three participants (Participants 1, 2 and 14) were 16 years old, one (Participant 11) was 15 years old, and one (Participant 13) was 14 years old.

In summary, the results showed positive and negative insights on what affected communication between professional nurses and high school learners. The negative perceptions of some participants could be addressed to foster effective communication in the school health programme.

## Discussion

The study objective was to describe the factors affecting HIV prevention communication between high school learners and professional nurses in eThekwini Metropolitan Municipality. From the study findings, it was found that communication is affected by the three main factors: trust between the high school learners and the school health nurse; communication is hindered by fear; and the frequency of visits to schools by the school health nurses affects communication negatively or positively.

The issue of trust affecting communication between healthcare providers and patients has also been described in several contexts outside South Africa. Wolderslund et al. (2021) in their study in Denmark note that elements of good patient-centred communication that improve the therapeutic relationships between healthcare providers and patients should have trust. Furthermore, an Italian study by Negro et al. (2020) provided an explanation of the issue of trust between healthcare workers and patients which resonates with these findings by highlighting that one of the determinants of trust in communication is the respect shown by the healthcare workers to the patients. This assertion of trust being built by respect described by Negro et al. (2020) is illustrated in this study by participants who noted that communication was good because they trusted the nurses who were polite and taught them about health-related issues.

The fear described by the participants emanated from the participants fearing the attitudes of the nurses. Similar conclusions were drawn from a study conducted in Fiji by Chandra and Mohammadnezhad (2021) who emphasised that rudeness, a lack of respectful communication, and non-responsiveness contribute to a negative demeanour. The issue of fear described by participants in this study also compares with the study conducted in Senegal by Adams et al. (2017) who noted that adolescents and young people feared communicating with healthcare workers because of fear associated with HIV stigma. Although there is a similarity in that fear is a barrier to communication, the difference in what the adolescents fear in this study (nurses’ attitudes) and the study conducted by Adams et al. (2017) (HIV stigma) illustrates how fear is a hindrance to communication that fosters the therapeutic relationship between care providers and adolescents and young people.

The participants noted that the nurses perceived the learners as too young to seek HIV testing services. On the other hand, learners perceived the nurses as old and this negatively affected the high school learners and professional nurses’ communication. Similar conclusions were drawn in another study conducted in South Africa by Bergam et al. (2022) who found that intergenerational pressures from healthcare stigmatised adolescents’ decisions on HIV prevention choices. Notably, the study findings regarding age differences between the nurses and the high school learners affecting communication are not unique to the South African context and validate findings from the USA by Zelazny et al. (2019) who noted that young women effectively communicate with healthcare workers who are at the same level as them and can be perceived as friends.

The infrequent visits by school nurses in the eThekwini Metropolitan Municipality affecting communication between high school learners and nurses also reflected on the issue of time affecting communication described by Zelazny et al. (2019). Zelazny et al. (2019) found that sensitive issues on reproductive health including HIV prevention need to be discussed in the middle of a healthcare visit. The issue of time in this study shows that the frequency of visits affected communication as infrequent visits of the healthcare workers affected communication between the high school learners and the professional nurses.

### Limitations

Every effort was made to ensure the accuracy of the study findings; however, limitations exist that affect the applicability of the study findings. The study sample was limited to two schools in the eThekwini Metropolitan Municipality, limiting the generalisation of study findings.

### Implications

The findings of the study imply that there is a need for nurses working in the school health programme to enhance patient-focussed communication with high school learners so that utilisation of HIV prevention services can be increased. For policymakers on school health programmes, there is a need to consider the intergenerational age differences that affect communication which result in stigmatisation of learners seeking HIV prevention services.

## Conclusion

From the study findings, it is therefore concluded that communication between high school learners and school nurses in the school health programme is determined by trust between the learners and the nurses, fear of the nurses working in the school health programme, age differences between the learners and the nurses, and the frequency with which visits occur in the schools for HIV prevention services. From these findings, it is recommended that nurses and policymakers improve patient-centred communication through improvement in the attitudes of nurses ensuring trust is gained without fear and assigning younger nurses to school health programmes to decrease intergenerational gaps. It is also recommended that school management should support visits by school health teams to foster trust and effective communication between nurses and learners. Furthermore, parents should encourage learners to communicate with nurses even though parents are familiar with the nurses from the community which would foster communication with adolescents mitigating the issue of fear.
